# Biofilm Formation on Endotracheal and Tracheostomy Tubing: A Systematic Review and Meta‐Analysis of Culture Data and Sampling Method

**DOI:** 10.1002/mbo3.70032

**Published:** 2025-07-07

**Authors:** Ed Deshmukh‐Reeves, Matthew Shaw, Charlotte Bilsby, Campbell W. Gourlay

**Affiliations:** ^1^ School of Biosciences, Division of Natural Sciences University of Kent Canterbury Kent UK

**Keywords:** biofilm, microbiome, Pseudomonas, respiratory infections

## Abstract

Biofilm formation on tracheal tubing is a key risk factor for ventilator‐associated pneumonia. Endotracheal tube microbiology has been systematically reviewed, but tracheostomy tube profiles have not. Analysis of the tube‐associated microbiome is not standardised, and sampling methods are varied. We compared the reported microbiomes of endotracheal and tracheostomy tubes and examined the impact of sampling by tracheal aspiration or direct culture. We searched PubMed, SCOPUS, and Web of Knowledge for clinical microbiology studies from 2000–2024, extracting tubing type, sampling method, and the most prevalent genera identified. Genera were compared by Spearman's rank correlation and pairwise analyses by Šidák's test. Extraction from 49 studies identified 30 genera. *Pseudomonas* was the most prevalent in all conditions followed by *Klebsiella, Staphylococcus*, and *Acinetobacter*. 25 studies performed tracheal aspiration, and 22, direct culture. Two studies used both methods. Correlation was observed between endotracheal and tracheostomy tubes, and aspirates and direct cultures (Spearman's rho = 0.69; 0.59). *Pseudomonas* were more prevalent in tracheostomy tubes (*p* < 0.0001). Coagulase‐positive *Staphylococci* were more common in tracheal aspirates, and coagulase‐negative *Staphylococci* in direct culture. The microbial profiles of endotracheal and tracheostomy tubes are comparable, with *Pseudomonas* being the most common coloniser. Our analyses suggest that tracheal aspiration can effectively identify the constituents of biofilms without requiring tube removal, making it a valuable tool for clinical researchers to analyse or monitor biofilms before extubation or device failure using existing microbiology procedures.

## Graphical abstract

This systematic review provides an up‐to‐date profile of tracheostomy and endotracheal tube microbiomes, with *Pseudomonas* emerging as the most prevalent genus overall. Microbial profiles were equivalent whether identified by tracheal aspiration or direct culture, supporting aspiration as a reliable, noninvasive approach for biofilm assessment without requiring extubation.

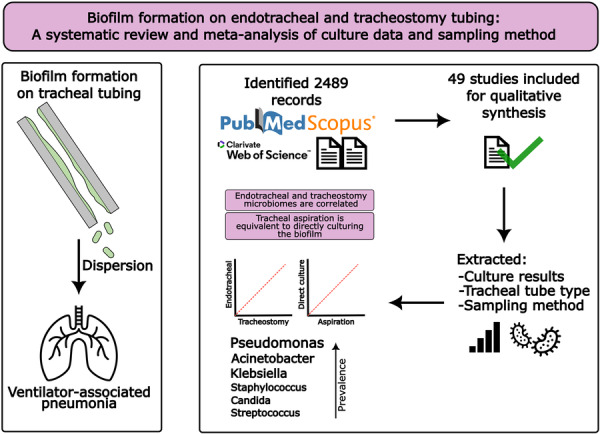

## 1 Introduction

Nosocomial infections particularly those caused by ESKAPE pathogens (*Enterococcus faecium, Staphylococcus aureus, Klebsiella pneumoniae, Acinetobacter baumannii, Pseudomonas aeruginosa, and Enterobacter spp*.), are associated with high morbidity and mortality. These pathogens are often resistant to multiple antibiotics, including last‐resort treatments (Kalpana et al. 2023; De Oliveira et al. 2020). A key risk factor for these infections, is the presence of medical devices, which are linked to 60‐70% of all nosocomial infections (Bouhrour et al. 2024). Indwelling devices such as urinary catheters and tracheal tubing, coated in bodily secretions, promote microbial proliferation and biofilm formation, offering enhanced protection from therapeutic interventions (El Cheikh et al. 2018; Sharma et al. 2023).

In clinical scenarios which the patient is unable to maintain a reliable passage of air, an artificial airway must be established via endotracheal or tracheostomy tube (Hickey et al. 2024). Biofilm formation on tracheal tubing is a major risk factor for ventilator‐associated pneumonia (VAP), which affects up to 40% of ventilated patients with a direct attributable mortality of approximately 10% (Papazian et al. 2020).

Biofilms are complex, polymicrobial communities of microorganisms adhered to a surface, and encased in an extracellular matrix of polysaccharides, nucleic acids and other biological material. This matrix enhances the biofilm's resistance to antimicrobial treatments, with the minimum inhibitory concentrations to treat biofilms, often hundreds of times higher than that for planktonic cells (Liu et al. 2022; Limoli et al. 2015; Nett et al. 2015).

Biofilm formation begins with planktonic cells adhering to a surface or substrate (Sharma et al. 2023). Surface‐attached cells proliferate and secrete substances that form the extracellular matrix. As the biofilm matures, it develops into 3D structures of cells and polymers (Arjes et al. 2021). Planktonic cells or whole aggregates originating from the biofilm can also dissociate, travelling to secondary locations, while retaining the antimicrobial tolerance traits observed in the surface‐attached biofilm (Chua et al. 2014). In tracheal tubing, the dissociation of cells and aggregates into tracheal secretions is crucial in the development of VAP (Papazian et al. 2020).

The key distinction between endotracheal tubes and tracheostomy tubes is their insertion route and resulting exposure to differing microbiomes. The endotracheal tube is inserted orally and therefore exposed to the oral microbiome while the tracheostomy bypasses the oral microbiome by insertion through a stoma in the neck. Research indicates that the oral microbiome may play a role in colonisation of the endotracheal tube and that good oral hygiene may prevent VAP (Santos Zambrano et al. 2024; Huang et al. 2025). However, a systematic review and meta‐analysis concluded that disinfection with chlorhexidine was ineffective at all concentrations in limiting VAP (De Cassai et al. 2024). This discordance suggests a more nuanced mechanism for the colonisation of tracheal tubing. Species known to cause VAP, such as *P. aeruginosa* and *S. aureus* are common nosocomial pathogens but not residents of the healthy oral flora suggesting environmental acquisition or dysbiosis (Fernández‐Barat et al. 2020). The precise mechanism by which colonisation of the tracheal tubing occurs and the pathogenesis of VAP remains unclear.

Complete characterisation of the microbiology associated with tracheal tubing is limited, and generally restricted to a single tubing type, with sampling methods often diverse. Commonly, clinicians will obtain microbial profiles upon extubation, cutting the distal tip of the tracheal tubing and performing microbiology assessments downstream (Cader et al. 2020; Ferreira et al. 2016). Another common method is by tracheal aspiration, in which sub‐glottic secretions are suctioned using a specialised suction tube, or by a secondary catheter inserted through the artificial airway. Suction can be performed above or below the cuff. While this technique does not retrieve a sample directly from the biofilm itself, it is easier to collect, as this suctioning is typically performed regularly to prevent accumulation and therefore does not require extubation (Sontakke et al. 2023).

To date, two systematic reviews (Mishra et al. 2024; Codru et al. 2024) have investigated biofilm formation on tracheal tubing, focusing exclusively on endotracheal tubes. While microbial characterisation was included in both, it was not the primary objective. Codru et al. summarised biofilm detection rates and their influence on microbiology, whereas Mishra et al. analysed strain‐specific biofilm formation rates and antibiotic resistance profiles.

This review aims to examine and compare the complete microbial profiles of endotracheal and tracheostomy tubing and analyse the differences between samples collected via tracheal aspiration and those directly from the biofilm. By including commensal microorganisms, our review aims to contextualise the causes of VAP within the microbiome, supporting future research into potential inter‐species interactions or microbiome‐based regulatory mechanisms. As no such systematic review has been published to date, this review aims to guide strategies against biofilm formation on tracheal tubing by characterising the microbiology and evaluating whether direct culture or aspiration sampling are suitable/comparable for identifying the constituents of tracheal tube biofilms and the potential causes of VAP.

## 2 Methods

### 2.1 Definitions

‘***Tracheal aspirates’***: Samples gathered by the suctioning of sub‐glottic tracheal secretions. By specialised endotracheal tube or secondary suction catheter. Can be performed above or below the cuff.

***‘Direct biofilm culture’***: The process of sampling from the surface of the tubing directly, generally requiring extubation and excision of a portion of tubing. This is followed by any microbiology process to dislodge a portion of the biofilm including swabbing, vortexing or sonicating and plate‐rolling.

### 2.2 Study Design

This systematic review was designed following the ‘Preferred Reporting Items for Systematic Reviews and Meta‐Analyses’ (PRISMA) guidelines (Page et al. 2021).

### 2.3 Search Strategy

PubMed, SCOPUS and WebOfKnowledge were searched for articles published between 1st January 2000 and 30th October 2024. The searches were performed using the Medical Subject Headings (MeSH) terms ‘tracheostomy’, ‘tracheotomy’, ‘biofilms’, ‘biofouling’, ‘isolation and purification’, ‘microbiology’, ‘pathogenicity’, ‘bacteria’, ‘fungi’, and ‘culture’, in addition to other relevant keywords. Keywords were connected using Boolean operators ‘AND’ and ‘OR’. Full details of the search are provided in the supporting material.

From each database, the retrieved record's titles and digital object identifier (DOI)(when available) were exported to a.CSV file, and duplicate records were removed. An initial screen of titles and abstracts was performed by E.D.R to remove irrelevant records. Records that passed the initial title/abstract screen were then exported to Mendeley Reference Manager (online version) for full text screening. Full text screening was performed independently by E.D.R, M.S and C.B in accordance with the defined eligibility criteria. Discordance was resolved by combined discussion until a consensus was reached.

### 2.4 Eligibility Criteria

For inclusion, studies were required to meet the following eligibility criteria: (1) Study included at least genus‐level culture data from patients intubated by endotracheal or tracheostomy tubing; (2) Culture data was grouped by the presence of either tracheostomy or endotracheal tubing; (3) Sample was collected by either tracheal aspiration, or direct culture from the tracheal tubing. Direct culture was defined as any form of swabbing/isolating from the tubing surface while still inserted, or removal of the tubing and any downstream culture‐based microbiology analysis.

Studies that met the following criteria were excluded: (1) studies that did not include genus level culture data from intubated patients; (2) studies that exclusively report culture data from specific, pre‐defined organism(s); (3) studies that combine/pool culture data from tracheostomy and endotracheal tubing; (4) Studies that combine/pool culture data from samples gathered by multiple methods; (5) studies that do not specify or use a sampling method other than tracheal aspiration or direct culture; (6) studies that perform exclusively molecular analyses; (7) in vitro or animal model investigations; (8) review articles.

In the full text screening process, the reason for exclusion was noted. Exclusion criteria (1) or (2) were marked as ‘Insufficient microbiology data’. Exclusion criteria (3) or (4) were marked as ‘Mixed sampling’ Exclusion criteria (5) were marked as ‘Insufficient sampling practice’.

### 2.5 Quality Assessment and Risk of Bias

Quality assessment of the included studies was performed using an adapted Newcastle‐Ottawa scale for cross‐sectional studies (Stang 2010). Each study was scored 0–9, with 0 being the highest risk of bias. Each study was scored for its sample size, representativeness, nonresponse rate, ascertainment of exposure, control of confounding variables, assessment of outcome, and use of statistical analysis. For this review, the primary confounding variables were the use of antibiotics before or during the study and the presence of a pre‐existing respiratory tract infection. Studies scoring 0‐3 were deemed very high risk for bias, 4‐6 as high risk for bias, and 7‐9 as low risk for bias.

### 2.6 Data Extraction and Ranking

Data from eligible studies was extracted into a Microsoft Excel spreadsheet with the pre‐defined headings of ‘Author’, ‘Date’, ‘Tubing Type’, ‘Organisms isolated’, ‘Number of organisms isolated’, and ‘Sampling method’. In the cases that a study had collected culture data from more than one point in time, the data associated with the final time point was extracted.

Once data was extracted it was processed into a summary by pooling number of organisms isolated by genus level. *Staphylococcus* was separated into Coagulase positive *Staphylococcus* (*CoPS*) and Coagulase negative *Staphylococcus* (*CoNS*). As *CoPS*, particularly *S. aureus*, are generally more associated with pathogenicity, and *CoNS* are often greater associated with biofilm formation/adhesion this distinction was of interest to investigate virulent/commensal biofilm formation. Any data referring to “Other organisms”, “Commensal organisms” or a taxa higher than genus level were omitted from the analysis. From this data a list of total detected genera was compiled.

All named genera were included to gain a comprehensive overview of the tracheal microbiome of intubated patients.

Per study, the genera were then ranked by abundance. Genera that occurred in the top 5 most abundant were scored 1‐5 (most abundant = 5) All other organisms were scored 0. Genera present on the list of total detected genera but not detected were also scored 0. In the cases of tied ranks, each tied genus was included in the summary. The mean rank for each organism was then calculated and compared across conditions.

## 3 Results

### 3.1 Study Selection

**Figure 1:**
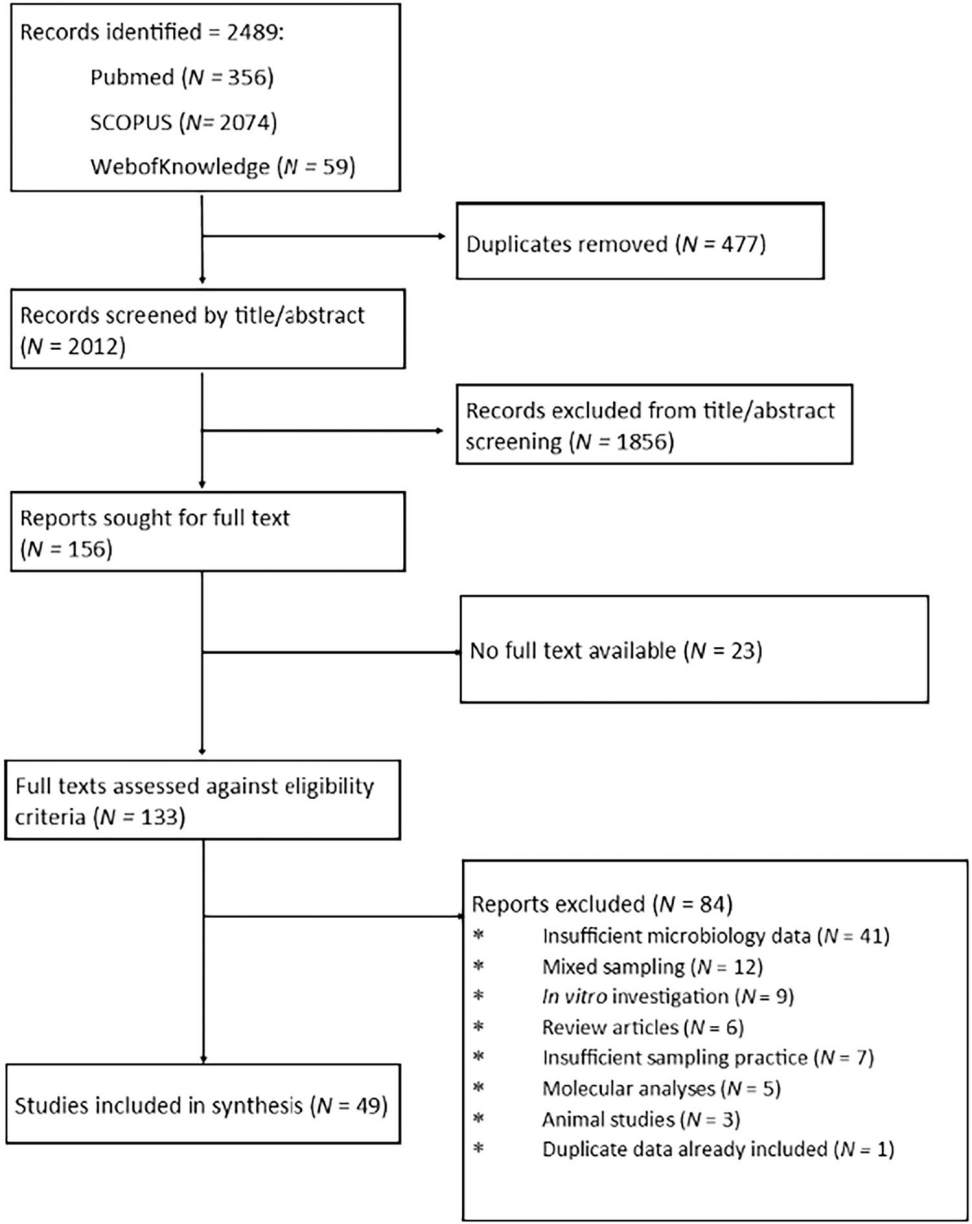
PRIMA flow of study selection process.

Study selection was performed in accordance with PRISMA guidelines. From the 3 databases searches performed 2489 records were identified, 2012 of which were unique. Following initial title/abstract screening 156 reports were sought for full text, of which 133 were accessed and screened against the eligibility criteria. 84 reports were excluded, with the most common cause for exclusion being ‘insufficient microbiology data’. While not specified in the eligibility criteria, two studies were excluded for the use of broncho‐alveolar lavage for to collect samples as our aim was to characterise the tracheal microbiome and the lower respiratory tract was deemed too distant. One study was excluded for reporting data already presented in another study included in the review. Forty nine studies aligned with the eligibility criteria and were progressed to data extraction (El Cheikh et al. 2018; Cader et al. 2020; Ferreira et al. 2016; Akrami et al. 2023; Aly et al. 2012; Bello et al. 2020; Chan et al. 2024; van Charante et al. 2022; Cifuentes et al. 2022; Danin et al. 2015; Dargahi et al. 2022; Duran et al. 2021; Fengling Yu et al. 2022; Friedland et al. 2001; García‐Boyano et al. 2023; Gil‐Perotin et al. 2012; Golli et al. 2019; Gupta et al. 2015; Hotterbeekx et al. 2016; Ismail et al. 2016; Jadhav and Deokar 2020; Khatri et al. 2023; Khosravi et al. 2012; Lee et al. 2012; Maldiney et al. 2022; McCaleb et al. 2016; McLaren et al. 2021; Mulla and Revdiwala 2011; Naderifar et al. 2024; Nagmoti et al. 2022; Phuaksaman et al. 2022; Pinheiro et al. 2021; Pons‐Tomàs et al. 2024; Raveendra et al. 2022; Sahoo et al. 2024; Saravanam et al. 2022; Ścibik et al. 2022; Shen et al. 2019; Shin et al. 2011; Singhai et al. 2012; Solomon et al. 2009; Taj et al. 2018; Thorarinsdottir et al. 2020; Tsukahara et al. 2022; Tuteja et al. 2022; Vandecandelaere et al. 2013, 2012; Vasconcellos Severo et al. 2023; Zorgani et al. 2015). The study selection process is summarised in Figure 1 and a list of the included studies and associated characteristics in Table 1.

**Table 1:** Summary of included studies and associated characteristics. ETT – Endotracheal tube, TT – Tracheostomy tube.

Author, year	Tubing type	Sampling method	Scoring genera	Citation
Akrami, 2023	Endotracheal	Direct	*Klebsiella, Escherichia, Pseudomonas, Citrobacter, Serratia, Acinetobacter*	Akrami et al. (2023)
Aly, 2012	Endotracheal	Aspiration	*Klebsiella, Pseudomonas, CoPS, CoNS, Streptococcus*	Aly et al. (2012))
Bello, 2020	Endotracheal	Aspiration	*Acinetobacter, CoPS, Pseudomonas, Klebsiella Escherichia*	Bello et al. (2020)
Cader, 2020	Tracheostomy	Direct	*Pseudomonas, Acinetobacter, Klebsiella, CoNS, Proteus, Escherichia, CoPS*	Cader et al. (2020)
Chan, 2024	Tracheostomy	Direct	*Stenotrophomonas, Corynebacterium, Streptococcus, Pseudomonas, CoNS, Acinetobacter, Neisseria, Klebsiella, CoPS, Enterococcus, Providencia, Achromobacter, Proteus, Micrococcus, Candida, Elizabethkingia*	Chan et al. (2024)
van Charante, 2022	Endotracheal	Direct	*Candida, CoNS, Streptococcus, Enterococcus, Klebsiella*	van Charante et al. (2022)
Cifuentes, 2022	Endotracheal	Direct	*Candida, Escherichia, Pseudomonas, Klebsiella, Nakaseomyces, Serratia, Stenotrophomonas, Moraxella, Raoultella, Citrobacter, Proteus, CoPS*	Cifuentes et al. (2022)
Danin, 2015	Endotracheal	Direct	*Pseudomonas, Streptococcus, CoNS, CoPS, Enterococcus, Candida*	Danin et al. (2015)
Dargahi, 2022	Endotracheal	Aspiration	*Streptococcus, Klebsiella, Pseudomonas, Acinetobacter, CoPS*	Dargahi et al. (2022)
Duran, 2021	Endotracheal	Aspiration	*Acinetobacter, Pseudomonas, Klebsiella, Escherichia, CoPS*	Duran et al. (2021)
El Cheikh, 2017	Tracheostomy	Aspiration	*Pseudomonas, Staphylococcus, Morganella, Klebsiella, Stenotrophomonas, Proteus*	El Cheikh et al. (2018)
Fengling Yu, 2022	Endotracheal	Aspiration	*Klebsiella, Pseudomonas, Acinetobacter, CoPS, Candida, Stenotrophomonas, Enterococcus*	Fengling Yu et al. (2022)
Ferreira, 2016	Endotracheal	Aspiration	*Pseudomonas, Streptococcus, Acinetobacter, CoNS, Klebsiella, Candida*	Ferreira et al. (2016)
Friedland, 2001	Endotracheal	Direct	*CoNS, Klebsiella, CoPS, Pseudomonas, Escherichia*	Friedland et al. (2001)
García‐Boyano, 2023	Tracheostomy	Aspiration	*Pseudomonas, CoPS, Serratia, Escherichia, Klebsiella*	García‐Boyano et al. (2023)
Gil‐Perotin, 2012	Endotracheal	Aspiration	*Candida, Acinetobacter, Pseudomonas, Staphylococcus*	Gil‐Perotin et al. (2012)
Golli, 2019	Endotracheal	Aspiration	*Klebsiella, CoPS, CoNS, Acinetobacter, Pseudomonas*	Golli et al. (2019)
Gupta, 2014	Endotracheal	Direct	*CoPS, Klebsiella, CoNS, Citrobacter, Micrococcus*	Gupta et al. (2015)
Hotterbeekx, 2016	Endotracheal	Direct	*Candida, CoNS, Nakaseomyces, Pseudomonas, Enterococcus, Escherichia*	Hotterbeekx et al. (2016)
Ismail, 2016	Endotracheal	Aspiration	*Klebsiella, Pseudomonas, CoNS, Candida, Enterobacter, Acinetobacter, Escherichia, CoPS, Serratia*	Ismail et al. (2016)
Jadhav & Deokar, 2020	Endotracheal	Aspiration	*Acinetobacter, Klebsiella, Pseudomonas, CoPS, Escherichia*	Jadhav and Deokar (2020)
Khatri, 2023	Endotracheal	Direct	*Acinetobacter, Klebsiella, Pseudomonas, Escherichia, CoPS*	Khatri et al. (2023)
Khosravi, 2012	Endotracheal	Direct	*Enterobacter, Pseudomonas, CoNS, CoPS, Escherichia, Proteus*	Khosravi et al. (2012)
Lee, 2012	Endotracheal	Aspiration	*Pseudomonas, CoPS, Acinetobacter*	Lee et al. (2012)
Maldiney, 2022	Endotracheal	Direct	*CoNS, Enterococcus, Candida, CoPS, Escherichia, Streptococcus*	Maldiney et al. (2022)
McCaleb, 2016	Tracheostomy	Aspiration	*CoPS, Pseudomonas, Stenotrophomonas, Serratia, Streptococcus*	McCaleb et al. (2016)
Mclaren, 2021	Tracheostomy	Aspiration	*CoPS, Pseudomonas, Haemophilus, Streptococcus, Klebsiella*	McLaren et al. (2021)
Mulla & Revdiwala, 2011	Both	Direct	*ETT: Pseudomonas, Acinetobacter, Klebsiella, Escherichia, Enterobacter TT: Pseudomonas, Acinetobacter, Klebsiella*	Mulla and Revdiwala (2011)
Naderifar, 2024	Endotracheal	Aspiration	*Klebsiella, Escherichia, Salmonella, Proteus*	Naderifar et al. (2024)
Nagmoti, 2022	Tracheostomy	Aspiration	*Pseudomonas, Klebsiella, Citrobacter, Enterococcus, CoPS*	Nagmoti et al. (2022)
Phuaksaman, 2022	Tracheostomy	Aspiration	*Pseudomonas, Moraxella, Acinetobacter, Klebsiella, CoPS*	Phuaksaman et al. (2022)
Pinheiro, 2021	Endotracheal	Aspiration	*Klebsiella, Candida, Acinetobacter, Enterobacter, CoPS, Chryseobacterium, Escherichia, Raoultella, Pseudomonas, Citrobacter*	Pinheiro et al. (2021)
Pons‐Tomàs, 2024	Tracheostomy	Aspiration	*Pseudomonas, CoPS, Moraxella, Streptococcus, Haemophilus*	Pons‐Tomàs et al. (2024)
Raveendra, 2022	Tracheostomy	Direct	*Klebsiella, Acinetobacter, Pseudomonas, CoPS, Escherichia*	Raveendra et al. (2022)
Sahoo, 2024	Tracheostomy	Aspiration	*Candida, Acinetobacter, Pseudomonas, Aspergillus, CoPS*	Sahoo et al. (2024)
Saravanam, 2022	Tracheostomy	Direct	*Pseudomonas, CoNS, Streptococcus, Escherichia, Klebsiella*	Saravanam et al. (2022)
Ścibik, 2022	Tracheostomy	Direct	*CoPS, Klebsiella, CoNS, Candida, Streptococcus, Escherichia, Enterobacter, Pseudomonas*	Ścibik et al. 2022
Shen, 2019	Endotracheal	Both	*Direct: Acinetobacter, Pseudomonas, CoPS, Klebsiella, Enterococcus Aspiration: Acinetobacter, Pseudomonas, Klebsiella, CoPS, Morganella, Proteus, Xanthomonas*	Shen et al. (2019)
Shin, 2011	Endotracheal	Aspiration	*Stenotrophomonas, CoPS, Pseudomonas, Acinetobacter*	Shin et al. (2011)
Singhai, 2012	Endotracheal	Direct	*Klebsiella, CoNS*	Singhai et al. (2012)
Solomon, 2009	Tracheostomy	Direct	*CoPS, CoNS, Escherichia, Pseudomonas, Streptococcus, Serratia, Stenotrophomonas, Proteus, Candida, Enterococcus*	Solomon et al. (2009)
Taj, 2018	Endotracheal	Direct	*Acinetobacter, Escherichia, Pseudomonas, Klebsiella*	Taj et al. (2018)
Thorarinsdottir, 2020	Endotracheal	Both	*Direct: Candida, Enterococcus, CoPS, Pseudomonas, Stenotrophomonas, Klebsiella, Chryseobacterium Aspirates: Candida, Enterococcus, CoPS, Stenotrophomonas, Pseudomonas, Haemophilus, Klebsiella*	Thorarinsdottir et al. (2020)
Tsukahara, 2022	Both	Aspiration	*ETT: Pseudomonas, Haemophilus, CoPS, Pandoraea, Escherichia, Aspergillus, Streptococcus TT: Pseudomonas, CoPS*	Tsukahara et al. (2022)
Tuteja, 2022	Endotracheal	Aspiration	*Acinetobacter, Klebsiella, Stenotrophomonas, Escherichia, Enterobacter, Elizabethkingia, Pseudomonas, CoNS, Achromobacter, Serratia*	Tuteja et al. (2022)
Vandecandelaere, 2012	Endotracheal	Direct	*CoNS, Micrococcus, Candida, CoPS, Klebsiella*	Vandecandelaere et al. (2012)
Vandecandelaere, 2013	Endotracheal	Direct	*CoNS, Micrococcus, CoPS, Candida, Klebsiella, Escherichia, Pseudomonas*	Vandecandelaere et al. (2013)
Vasconcellos Severo, 2023	Tracheostomy	Aspiration	*Pseudomonas, CoPS, Stenotrophomonas, Klebsiella, Morganella, Serratia, Proteus, Streptococcus, Acinetobacter, Citrobacter, Haemophilus*	Vasconcellos Severo et al. (2023)
Zorgani, 2015	Both	Direct	*ETT: Klebsiella, Acinetobacter, Pseudomonas, Enterobacter, Serratia, Proteus TT: Klebsiella, Pseudomonas*	Zorgani et al. (2015)

### 3.2 Quality Assessment

Across the 49 assessed studies, the mean scoring based on the Newcastle‐Ottawa scale was 6.9. Primarily, studies were deducted points based on the control of confounding variables, in which 39 studies scored 0. The absence of statistical analysis led to a 1‐point deduction in 11 studies, and a small sample size was recorded in 5 studies. In summary, 38 studies were scored as low risk for bias, and 11 as high risk of bias. Of the studies scored as high risk for bias, 6 would be scored as low risk, if not considering the use of statistical analysis, which, in the context of our review, is not essential for the unbiased extraction and analysis of raw data. The complete scoring is available in the supporting material.

### 3.3 Study Characteristics

Data was extracted from 31 studies investigating exclusively endotracheal tubing, and 15 investigating tracheostomy tubing. 3 studies independently analysed both endotracheal and tracheostomy tubes, totalling 49 studies. 34/49 studies analysed endotracheal tubing. Of these, 16 studies performed tracheal aspirates and 16 performed direct culture. 2 studies performed both sampling methods. 18/49 studies analysed tracheostomy tubing. Of these, 10 performed tracheal aspirates, and 8 performed direct culture. Tables ranking the prevalence of each genus by tubing type and sampling method are summarised in the complete data extraction, included in the supporting material.

**Figure 2:**
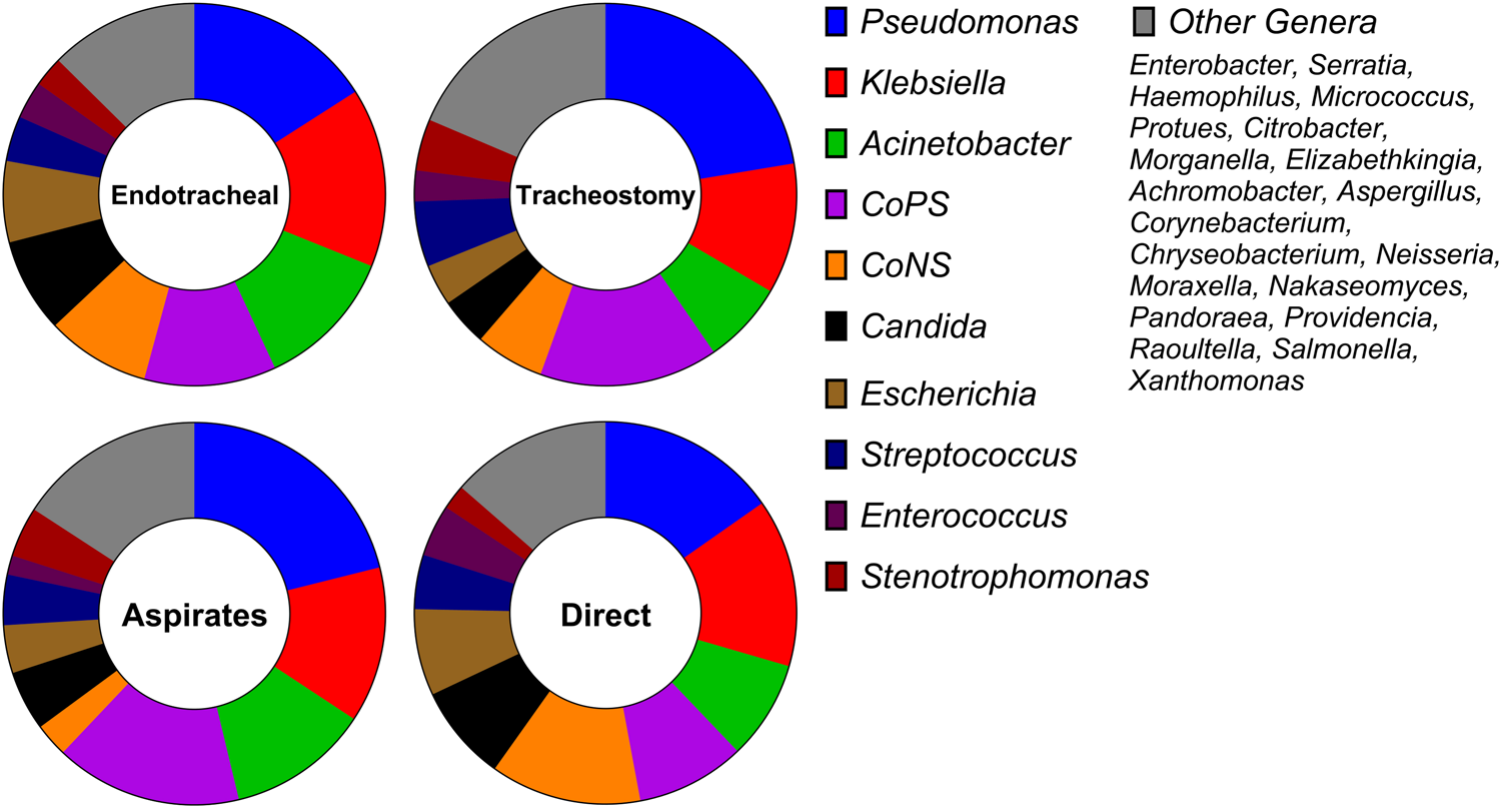
Prevalence of genera in endotracheal and tracheostomy tubing when sampled by tracheal aspiration or direct culture. Proportions of most prevalent isolates identified by all sampling methods from (upper‐left) endotracheal tubes or (upper‐right) tracheostomy tubes. Summary of most prevalent isolates identified on both tracheostomy and endotracheal tubes samples by (lower‐left) tracheal aspiration or (lower‐right) direct culture. A breakdown of prevalence of “Other Genera” is available in the supplementary material (CoPS: *Coagulase positive Staphylococcus*, “CoNS”: *Coagulase negative Staphylococcus*).

25 genera were identified across the 49 studies. In all groups, *Pseudomonas* was the most commonly identified genus, with a combination of *Klebsiella, Staphylococcus* and *Acinetobacter* forming the majority of detected isolates. *Candida spp*. were the most abundant fungal pathogen and the most prevalent genus after the 4 most common bacteria (Figure 2).

**Figure 3:**
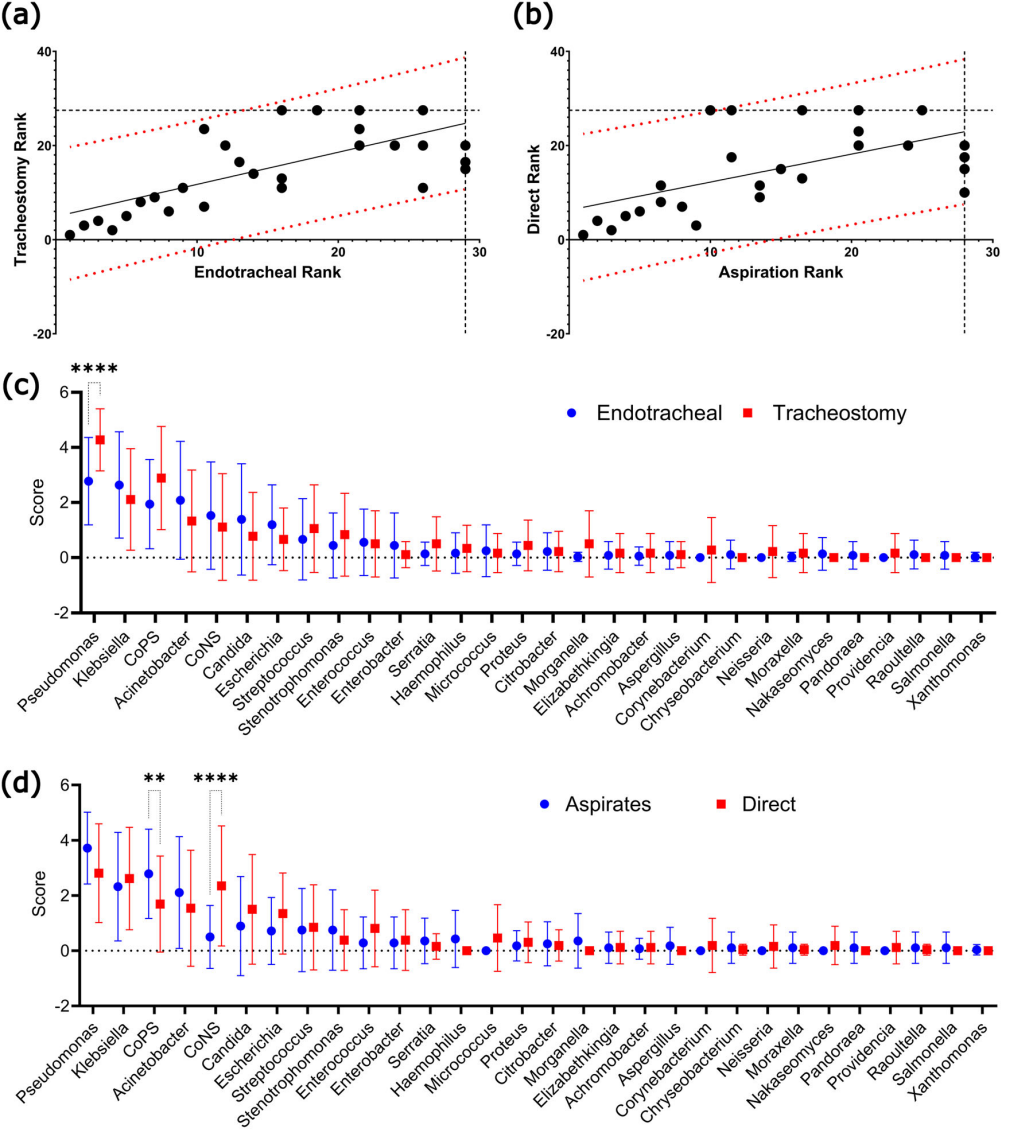
Meta‐analysis of extracted data. Genera were ranked by prevalence within each study and then scored based on their mean rank. Spearman's rank correlation of (a) endotracheal vs tracheostomy tubes, (Spearman's rho = 0.61, *p* < 0.0001) and (b) aspirate vs direct samples (Spearman's rho = 0.59, *p* < 0.001). Pairwise comparisons of mean score of (c) endotracheal vs tracheostomy tubes, and (d) aspirate vs direct samples. “CoPS”: Coagulase positive Staphylococcus, “CoNS”: Coagulase negative Staphylococcus (Statistical significance calculated by way of Šidák's multiple comparisons test ****p < 0.0001, **p < 0.01).

Good correlation was observed between both endotracheal and tracheostomy samples, as well as samples collected by either direct sampling, or tracheal aspiration. (Spearman's rho = 0.69 and 0.59 respectively). To calculate variance of individual genera, we performed pairwise comparisons of each genus' mean score. Our analysis showed that *Pseudomonas* was significantly more prevalent in tracheostomy tubes than in endotracheal tubes (*p* < 0.0001). For sampling method, the only significant difference was between *CoPS* and *CoNS*, with more *CoNS* found in direct cultures (*p* < 0.0001) and more *CoPS* in aspirate samples (*p* < 0.01) (Figure 3).

Our review included three studies that sampled both tracheostomy and endotracheal tubing, and two that compared tracheal aspiration with direct culture, enabling comparisons while controlling for extraneous variables. Of the studies comparing tubing types, only Tsukahara et al. addressed concordance, finding good agreement but limited conclusions due to a small sample size. Both studies comparing aspiration and direct culture found concordance, with Thorarinsdottir et al. noting that early surveillance cultures often predicting extubation cultures. A summary of these studies is provided in Table 2.

**Table 2:** Summary of independent comparisons. Studies including both endotracheal and tracheostomy tubes, or aspiration and direct culture samples.

Author, citation	Comparison	Conclusion
Mulla and Revdiwala (2011)	Endotracheal vs Tracheostomy	Not addressed by authors. Higher genus diversity in endotracheal samples. Concordance between most prevalent genera.
Tsukahara et al. (2022)	Endotracheal vs Tracheostomy	Sample size of tracheostomy tubes too small for viable comparison. Greater proportion of *Pseudomonas* in tracheostomy samples.
Zorgani et al. (2015)	Endotracheal vs Tracheostomy	Not directly addressed by authors. Some concordance, with higher prevalence of *Pseudomonas* in tracheostomy samples.
Shen et al. (2019)	Aspiration vs Direct	Strong correlation between samples collected by aspiration and direct culture.
Thorarinsdottir et al. (2020)	Aspiration vs Direct	Endotracheal aspirate surveillance cultures were often predictive of direct culture on extubation. Concordance between most prevalent genera.

## 4 Discussion

This review analyses clinical microbiology data on biofilm formation on endotracheal and tracheostomy tubes. Consistent with previous systematic reviews on endotracheal tubes, *Pseudomonas* was the most common genus on both tubing types, followed by *Klebsiella, Staphylococcus* and *Acinetobacter*. A slight increase in prevalence of *Pseudomonas* was observed on tracheostomy tubes. This is possibly due to their use in long‐term intubation, which may allow *Pseudomonas* to outcompete other biofilm contributors, in line with current literature trends (Cheng et al. 2019; Ammann et al. 2016). The microbial profiles of both tube types otherwise showed good concordance with only minor changes in ranks.

Tracheal aspiration samples also correlated well with direct biofilm cultures. The only significant difference was within the *Staphylococcus* genus, in which *CoPS* were more common in aspirates and *CoNS* were more common in biofilms. This may indicate increased dissociation of *CoPS* or enhanced adhesion of *CoNS*. There may also be a bias in studies collecting aspirate samples for patients diagnosed or suspected of pneumonia, therefore skewing the data towards pathogenic organisms and causes of VAP such as *Staphylococcus aureus*.

ESKAPE pathogens were highly prevalent among tracheal tube isolates, representing the four most commonly detected. This indicates that tracheal tube biofilms are likely to contain multidrug‐resistant strains (Kalpana et al. 2023; De Oliveira et al. 2020). *Candida spp*. were also frequently identified. This is of interest as there are numerous studies reporting synergism between the *Candida spp*. and each of the four most common bacterial pathogens identified in this study (Chen et al. 2014; Tan et al. 2016; Kong et al. 2016; Papadimitriou‐Olivgeris et al. 2014).

We acknowledge this review has some limitations, notably the inclusion of studies with variable inclusion criteria. Some studies collected data from all intubated patients, while others focussed only on those with a pneumonia diagnosis, which as mentioned above, may skew our results towards common causes of VAP. Timing of collection, patient age, and the precise culture conditions were also varied or omitted from the methods sections of some studies. VAP has a global burden, so our analysis aimed to compile data from the greatest range of studies without compromising the quality of the synthesis. By controlling each of these variables too stringently our analyses would exclude studies disproportionately from studies conducted in lower‐middle income countries and prevent the completion of a comprehensive review. Another key limitation was that our study was performed at the genus level, limiting the distinction between some pathogens and commensals of the same genus. The study was designed this way to better represent genera that were highly prevalent, but split across many species, such as *Candida* and *Streptococcus*.

This review shows good concordance between the microbial profiles of endotracheal and tracheostomy tubing. Recent research highlights a possible role of the oral microbiome in tubing colonisation, particularly *Pseudomonas, Klebsiella*, and *Staphylococcus* which are associated with oral dysbiosis and disease (Zuanazzi et al. 2010; Takahama Jr et al. 2021). Our analysis, comparing both orally and inter‐tracheally inserted tubing, finds minimal differences in the microorganisms colonising the devices. This suggests that the oral cavity may not be the primary source of biofilm‐forming pathogens, supported by the inefficacy of oral disinfection in preventing the onset of VAP (De Cassai et al. 2024).

The microbial profile of both commensal and VAP‐causing genera was comparable between samples collected by aspiration and those collected directly from the biofilm, besides an increased prevalence of *CoPS* in aspirates, and *CoNS* in the biofilm. This suggests that routine tracheal aspiration may be sufficient for detection of biofilm‐related VAP‐causing pathogens. Detection of pathogenic organisms in routine aspirates could be utilised as an indication to replace current tubing or begin treatment with prophylactic antimicrobials before the development of pneumonia.

Additionally, as tracheal aspiration is already a standard care practice, it offers a practical tool for clinical research, examining both the presence of VAP‐causing pathogens, or the complete microbiome. Aspiration allows for continuous monitoring of biofilm colonisation without requiring extubation, saving time and costs by leveraging existing microbiological workflows.

## Author Contributions

**Ed Deshmukh‐Reeves:** conceptualisation, investigation, writing – original draft, methodology, validation, visualisation, writing – review and editing, formal analysis, data curation. **Matthew Shaw:** writing – review and editing, investigation, data curation. **Charlotte Bilsby:** writing – review and editing, investigation, data curation. **Campbell W. Gourlay:** funding acquisition, writing – review and editing, project administration, resources, supervision.

## Ethics Statement

The authors have nothing to report.

## Conflicts of Interest

The authors declare that funding to EDR to support his PhD studentship as part of an industry CASE studentship was provided by ICU Medical Inc. However, no member of ICU Medical played any part in the research that contributed to this publication or in its writing or editing before submission. This article was not commissioned nor conceived by ICU Medical.

## Supporting information

Extracted Data gh.

Newcastle‐Ottawa Assessment gfg.

prisma checklist.

Search terms.

## Data Availability

The data that support the findings of this study are available from the corresponding author upon reasonable request.
