# Supplementary material for: Biofilm Formation on Endotracheal and Tracheostomy Tubing: A Systematic Review and Meta‐Analysis of Culture Data and Sampling Method
Source: Microbiologyopen. 2025 Jul 7;14(4):e70032. doi: 10.1002/mbo3.70032 (PMC12230368; doi:10.1002/mbo3.70032)
Supplement: Supplementary file 4 — Search terms. [file MBO3-14-e70032-s002.pdf]

PubMed Search

#1 57,269 results

((tracheostomy) OR (tracheotomy)) OR (endotracheal)

"tracheostomy"[MeSH Terms] OR "tracheostomy"[All Fields] OR "tracheostomies"[All Fields] OR "tracheotomy"[MeSH Terms] OR "tracheotomy"[All Fields] OR "tracheotomies"[All Fields] OR "endotracheal"[All Fields] OR "endotracheally"[All Fields]

#2 243,512 results

(tube) OR (tubing)

"tube"[All Fields] OR "tube s"[All Fields] OR "tubed"[All Fields] OR "tubes"[All Fields] OR "tubing"[All Fields] OR "tubings"[All Fields]

#3 5,214,680 results

(((((biofilm) OR (biofouling)) OR (characterise)) OR (characterize)) OR (isolate)) OR (profile)

"biofilm s"[All Fields] OR "biofilmed"[All Fields] OR "biofilms"[MeSH Terms] OR "biofilms"[All Fields] OR "biofilm"[All Fields] OR ("biofoulant"[All Fields] OR "biofoulants"[All Fields] OR "biofouled"[All Fields] OR "biofouler"[All Fields] OR "biofoulers"[All Fields] OR "biofouling"[MeSH Terms] OR "biofouling"[All Fields]) OR ("characterisation"[All Fields] OR "characterisations"[All Fields] OR "characterise"[All Fields] OR "characterised"[All Fields] OR "characterises"[All Fields] OR "characterising"[All Fields] OR "characterization"[All Fields] OR "characterizations"[All Fields] OR "characterize"[All Fields] OR "characterized"[All Fields] OR "characterizes"[All Fields] OR "characterizing"[All Fields]) OR ("characterisation"[All Fields] OR "characterisations"[All Fields] OR "characterise"[All Fields] OR "characterised"[All Fields] OR "characterises"[All Fields] OR "characterising"[All Fields] OR "characterization"[All Fields] OR "characterizations"[All Fields] OR "characterize"[All Fields] OR "characterized"[All Fields] OR "characterizes"[All Fields] OR "characterizing"[All Fields]) OR ("isolate"[All Fields] OR "isolate s"[All Fields] OR "isolated"[All Fields] OR "isolates"[All Fields] OR "isolating"[All Fields] OR "isolation and purification"[MeSH Subheading] OR ("isolation"[All Fields] AND "purification"[All Fields]) OR "isolation and purification"[All Fields] OR "isolation"[All Fields] OR "isolations"[All Fields]) OR ("profile"[All Fields] OR "profiled"[All Fields] OR "profiler"[All Fields] OR "profilers"[All Fields] OR "profiles"[All Fields] OR "profiling"[All Fields] OR "profilings"[All Fields])

#4 5,291,037 results

(((((microbiology) OR (microorganism)) OR (pathogen)) OR (bacteria)) OR (fungi)) OR (culture)

"microbiology"[MeSH Subheading] OR "microbiology"[All Fields] OR "microbiology"[MeSH Terms] OR "microbiology s"[All Fields] OR "microorganism"[All Fields] OR "microorganism s"[All Fields] OR "microorganisms"[All Fields] OR "pathogen"[All Fields] OR "pathogen s"[All Fields] OR "pathogene"[All Fields] OR "pathogenes"[All Fields] OR "pathogenic"[All Fields] OR "pathogenically"[All Fields] OR "pathogenicities"[All Fields] OR "pathogenicity"[MeSH Subheading] OR "pathogenicity"[All Fields] OR "virulence"[MeSH Terms] OR "virulence"[All Fields] OR "pathogenity"[All Fields] OR "pathogenous"[All Fields] OR "pathogens"[All Fields] OR "bacteria s"[All Fields] OR "bacteriae"[All Fields] OR "bacterias"[All Fields] OR "microbiology"[MeSH Subheading] OR "microbiology"[All Fields] OR "bacteria"[All Fields] OR "bacteria"[MeSH Terms] OR "fungi"[MeSH Terms] OR "fungi"[All Fields] OR "fungus"[All Fields]

OR "fungis"[All Fields] OR "microbiology"[MeSH Subheading] OR "microbiology"[All Fields] OR "culturabilities"[All Fields] OR "culturability"[All Fields] OR "culturable"[All Fields] OR "culturalism"[All Fields] OR "culture"[MeSH Terms] OR "culture"[All Fields] OR "cultural"[All Fields] OR "culturally"[All Fields] OR "cultures"[All Fields] OR "culture s"[All Fields] OR "cultured"[All Fields] OR "culturing"[All Fields] OR "culturings"[All Fields] OR "ethnology"[MeSH Subheading] OR "ethnology"[All Fields]

#6 3,408,794 results

review[publication type]

"review"[Publication Type]

#7 24,349,101 results

(2000/01/01:3000/12/12[Date - Publication])

2000/01/01:3000/12/12[Date - Publication]

#8 356 results

#1 AND #2 AND #3 AND #4 NOT #6 AND #7

SCOPUS Search

(ALL (tracheostomy OR tracheotomy) AND ALL (tube OR tubing) AND ALL (biofilm OR biofouling OR biofilm AND growth))

2,074 results

Web Of Knowledge Search

((ALL=(Tracheostomy OR Tracheotomy)) AND ALL=(Biofilm OR Biofilm growth OR Biofouling)) AND ALL=(tube OR tubing)

59 Results

Total 2489
